# A Simple and Convenient Synthesis of Unlabeled and ^13^C-Labeled 3-(3-Hydroxyphenyl)-3-Hydroxypropionic Acid and Its Quantification in Human Urine Samples

**DOI:** 10.3390/metabo8040080

**Published:** 2018-11-21

**Authors:** Yeganeh Khaniani, Matthias Lipfert, Dipanjan Bhattacharyya, Rolando Perez Pineiro, Jiamin Zheng, David S. Wishart

**Affiliations:** 1 Departments of Biological Sciences, University of Alberta, Edmonton, AB T6G 2E8, Canada; khaniani@ualberta.ca (Y.K.); lipfert@ualberta.ca (M.L.); dbhattac@ualberta.ca (D.B.); rpp_10400@yahoo.com (R.P.P.); jiamin3@ualberta.ca (J.Z.); 2 Departments of Computing Sciences, University of Alberta, Edmonton, AB T6G 2E8, Canada; 3 National Institute of Nanotechnology, 11421 Saskatchewan Drive, Edmonton, AB T6G 2M9, Canada

**Keywords:** HPHPA, biomarker, isotopic labeling, sonication, Reformatsky reaction

## Abstract

An improved method to synthesize the highly abundant and biomedically important urinary metabolite 3-(3-hydroxyphenyl)-3-hydroxypropionic acid (HPHPA) is reported. The modified protocol is based on an indium-mediated sonochemical Reformatsky reaction. The synthesis is a simple two-step route as opposed to a complex four-step route previously reported in the literature that requires specialized equipment, flammable materials, and high-pressure reaction vessels. The described procedure also provides an expedient route to prepare a ^13^C isotopically labeled HPHPA that can be used as a standard for quantitative LC-MS analysis. This report also illustrates how the synthesized metabolite standard was used to detect and accurately quantify its presence in human urine samples using both NMR and LC-MS techniques.

## 1. Introduction

3-(3-Hydroxyphenyl)-3-hydroxypropionic acid (HPHPA) is a highly abundant (10–1000 M) but little-studied metabolite normally found in human urine [1,2]. It is believed to be formed from the action of specific gut microflora (especially *Clostridia* sp.) on polyphenolic compounds found in fruits [3]. The presence of abnormal concentrations of HPHPA in body fluids has been associated with gut dysbiosis as well as several neurological conditions, including autism and schizophrenia [4]. It has been suggested that HPHPA may act as a dopamine or catecholamine analog. Excess dopamine and dopamine-like compounds have been linked to abnormal or psychotic behavior [5]. As a result, HPHPA has been touted as a possible diagnostic biomarker for several neurological conditions [6]. 

For many years, our group has been working on the systematic identification, quantification, and annotation of human metabolites in biological fluids [1,2]. As part of this effort, we have synthesized many commercially unavailable metabolites and have been using them as reference compounds to confirm their existence or to quantify their abundance in various biofluids [7]. Given the potential importance of HPHPA as a disease biomarker, the lack of commercially available sources, and the paucity of existing literature regarding its synthesis, we were led to explore new ways to synthesize this metabolite and its stable isotope-labeled version.

The original report on the synthesis of HPHPA [8] involved four steps, including a protection–deprotection sequence. Here we report a much simpler and more efficient route that does not require the use of any protecting group. Key to this simplified approach is the use of an indium-mediated Reformatsky reaction. Several modified Reformatsky reactions using different metals have been described previously [9,10,11,12,13,14]. Some of these use special activation techniques such as acid washing, which removes the oxide layer from the surface and forms a finely dispersed metal for in situ reduction [15]. However, in the year 2000, P.H. Lee et al. reported an indium-mediated Reformatsky reaction [16] that has some significant advantages over other metal catalysts such as zinc, tin, bismuth, lead, or cadmium. These advantages include easy handling, high reactivity, high selectivity, and low toxicity [17,18,19]. Recently, significant rate enhancements of these Reformatsky reactions have been achieved when carried out in the presence of ultrasonic waves generated by bench-top sonicators [20]. The main advantages of ultrasonic activation are the short reaction time and the high yield. The approach described here uses both an indium-based Reformatsky reaction and ultrasonic activation to synthesize both unlabeled and ^13^C-labeled HPHPA. We also describe how these HPHPA standards were used to create reference spectra and reference MRM (multiple reaction monitoring) transitions for the detection and quantification of HPHPA in human urine by semi-automated NMR and LC-MS/MS-based metabolomic techniques.

## 2. Results and Discussion

### 2.1. Synthesis of HPHPA

**Scheme 1:**
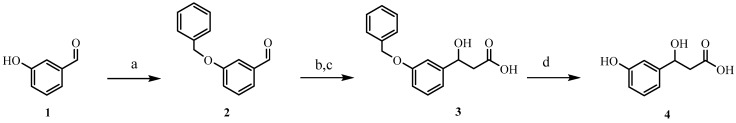
Reagents and conditions: (a) benzyl chloride, K_2_CO_3_, EtOH, 70 °C, 12 h, 100%; (b) Zn, ethyl bromoacetate, benzene, 80 °C, 4 h, 79%; (c) NaOH (2 N)/MeOH, 20 min, then HCl (2 N), 100%; (d) Pd-C/ H_2_, MeOH, 12 h, 80%.

Our initial attempts to synthesize HPHPA, which were based on the method described in 1957 [8] with slight modification, started with the initial protection of the phenolic hydroxyl of the starting 3-hydroxybenzaldehyde **1** using a benzyl group. Next, the protected benzaldehyde **2** was subjected to a Reformatsky reaction in the presence of zinc and ethyl bromoacetate to afford the hydracrylic ester, which was then hydrolyzed to the corresponding carboxylic acid derivative **3**. In the last step of the synthesis, the benzyloxy group in **3** was removed by using catalytic hydrogenation in the presence of Pd-C under an H_2_ atmosphere to produce HPHPA **4** (see Scheme 1a–d). We repeated the above HPHPA synthesis as described multiple times and, while it was successful, we never achieved a final yield greater than 63% as opposed to the reported overall yield of 79%. We suspect the limited analytical tools available in 1957 (i.e., no NMR) along with the use of crude crystallization methods likely led to an overestimation of the reported yield. Because of the modest yield as well as the difficulty and time-consuming nature of the published HPHPA synthesis, we decided to explore the development of a more facile, higher-yielding approach that would also enable the preparation of a ^13^C-labeled HPHPA.

**Scheme 2:**
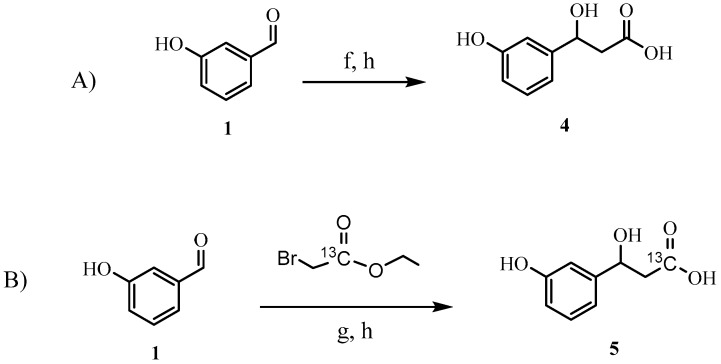
(**A**) Synthesis of unlabeled 3-(3-hydroxyphenyl)-3-hydroxypropionic acid (HPHPA). (**B**) Synthesis of labeled HPHPA. Reagents and conditions: (f) In, ultrasound, ethyl bromoacetate, THF, 70%; (g) In, ultrasound, (1-^13^C, 99%) ethyl bromoacetate, THF, 70%; (h) NaOH (2 N)/MeOH, 20 min, then HCl (2 N), 100%.

Using the method described by Lee et al. [16] and incorporating ultrasonic activation, we were able to develop a far simpler, higher yielding, two-step procedure for the direct synthesis of HPHPA starting from 3-hydroxybenzaldehyde **1** without the need to protect the hydroxyl group (Scheme 2A). This modified protection-free procedure generated the product in 70% yield after saponification.

A particularly appealing aspect of sonication-promoted Reformatsky reactions is that they can be run at room temperature under neutral conditions over a short reaction period. Avoiding moisture through the use of dry solvents and a nitrogen atmosphere was found to improve the overall reaction efficiency. However, the yield decreased when lower-frequency/lower-power sonicators were used under identical conditions.

This modified protocol was also used to synthesize ^13^C-labeled HPHPA (Scheme 2B). To this end, we used ^13^C-labeled ethyl bromoacetate (1-^13^C, 99%), which was labeled at the carbonyl position. This compound was used as a coupling partner in the Reformatsky reaction with hydroxybenzaldehyde **1**. As with the previously described Reformatsky procedure, the ^13^C-hydracrylic ester was hydrolyzed to yield the ^13^C-labeled HPHPA **5**. The structure of **5** was confirmed by NMR spectroscopy (at 700 MHz), and the presence of the ^13^C isotopic label was confirmed by HRMS (high-resolution mass spectrometry), as well as by ^13^C-NMR and 2D NMR experiments.

The indium-mediated Reformatsky reaction was also attempted without sonication. Indium powder was washed with aqueous 2 N HCl and then dried in an oven for 12 h. The activated indium powder was then used for the reaction. A stepwise procedure was used where indium powder and ethyl bromoacetate were first taken in dry THF and stirred for 5 h at room temperature while the required organometallic intermediate was generated. A solution of the starting aldehyde **1** was then added, and the reaction was continued under argon for 16 h. After work-up, the crude mixture was hydrolyzed as before, followed by purification. This led to 65% yield of the final product, which was comparable to the sonication procedure. As expected, the sonication approach had a much shorter reaction time.

### 2.2. Assay Validation-HPHPA

The concentration of HPHPA in 10 human urine samples was measured by NMR and LC-MS/MS techniques using the synthesized pure HPHPA as a reference standard. A comparative study of the results obtained by those two methods was also conducted. Chemical standards including HPHPA were all accurately weighed and fully dissolved in water to provide the necessary calibration curve solutions. All solutions were stored at −80 °C until required. Quantification of HPHPA by LC-MS/MS was done by comparing ratios of the peak area of unlabeled HPHPA to its corresponding ^13^C-labeled internal standard (i.e., ^13^C-HPHPA). Good calibration regression of HPHPA was established over a calibration range from 5 to 400 µM, with an *R* = 0.9999. The LOD (limit of detection) and LOQ (limit of quantitation) of HPHPA were 0.005 and 0.0167 µM, respectively. No significant carry-over was observed in the assay. Inter-day and intra-day accuracy and precision were evaluated by analyzing previously prepared synthetic mixtures in triplicate, at three different concentration levels on three different days. Our results indicate that the developed LC-MS/MS method is able to quantify HPHPA in an accurate, robust, and reproducible manner, as judged by the following two criteria: (1) accuracy within 100% ± 10%; and (2) precision within 10%. The recovery of the measured HPHPA from a commercial pooled human urine sample was checked by comparing the calculated spiked concentration with the fortified amount at three different concentration levels. The calculated recoveries were 96.4%, 97.5%, and 104.4% for the low, medium, and high spike-in concentrations, which were all within our defined acceptance criteria.

### 2.3. LC-MS/MS Assay Validation of HPHPA on Random Urine Samples

Ten random human urine samples were tested for the presence of HPHPA to further validate our assay. All samples were found to have detectable levels of HPHPA. The calculated concentration of HPHPA in these samples ranged from 0.995 to 16.6 µM/mM creatinine. These values are all within the reported normal reference values [2].

### 2.4. Cross-Validation with NMR on Human Urine Samples

**Figure 1:**
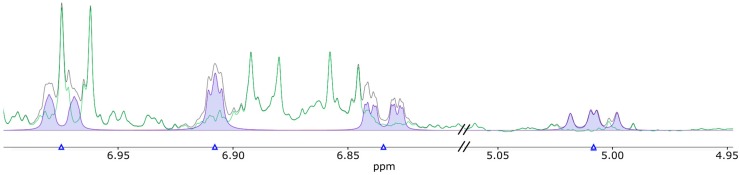
Extract of a profiled NMR spectrum of a typical human urine sample using Chenomx software suite. The black line represents the actual urine spectrum, while the green line represents the difference between the actual spectrum and the fitted spectrum. Highlighted in blue are the clusters of HPHPA between 7.00 and 6.80 ppm and the cluster at 5.00 ppm.

To cross-validate the LC-MS/MS assay, the above 10 human urine samples were also measured by NMR, and the obtained results were compared. The Chenomx NMR Suite software (version 8.3 Chenomx, Inc., Edmonton, AB, Canada) was used to generate a spectral library entry for HPHPA with the reference spectrum recorded as described in the Materials and Methods. HPHPA was identified and quantified in all urine samples using the new HPHPA spectral library entry. Figure 1 shows an example of how HPHPA was measured in a typical human urine sample. The signals at 5.0 and 6.8 ppm were used for quantification since they are in a relatively clear spectral region.

Table 1 summarizes the data comparison between the HPHPA data collected on an LC-MS/MS platform and our NMR platform. HPHPA concentration differences obtained by the two different methods were calculated to vary from 0.13% to 7.69%, which are all within the acceptable ranges.

## 3. Materials and Methods

### 3.1. Materials and Methods for HPHPA Synthesis

3-Hydroxybenzaldehyde, ethyl bromoacetate, and indium metal powder were obtained from Sigma-Aldrich (St. Louis, MO, USA). Ethyl bromoacetate-1-^13^C was purchased from CDN Isotopes (Quebec, QC, Canada). Ultrasonication reactions were carried out in a Model FS30 Fisher Scientific ultrasonic cleaner bath (Pittsburg, PA, USA) which delivered an ultrasonic frequency of 42 kHz at 100 W. NMR spectra were recorded on a Bruker Avance III HD (700 MHz) spectrometer operated at 700.32 MHz ^1^H resonance frequency (Bruker Biospin, Rheinstetten, Germany). HRMS spectra of the pure HPHPA standards were recorded on an Agilent 6220 time of flight (TOF) mass spectrometer (Palo Alto, CA, USA) in the negative ion mode using an ESI (electrospray ionization) interface. The HPLC chromatograms for the HPHPA samples were collected on an Agilent 1100 series HPLC system equipped with a 250 mm × 4.6 mm C3 Agilent column (5 µm particle size) using UV detection wavelengths of 210 and 254 nm.

The synthesis of HPHPA was performed using the following protocol. To a solution of indium powder (950 mg, 8.3 mmol) in dry THF (16 mL) was added 3-hydroxybenzaldehyde (1 g, 8.2 mmol) and ethyl bromoacetate (2.1 g, 1.4 mL, 12.3 mmol) under nitrogen at room temperature. After 2.5 h of sonication (at 42 kHz, 100 W), the reaction was quenched with NH_4_Cl and extracted with EtOAc (3 × 25 mL). The solvent was removed in vacuo. The residue was dissolved in MeOH/water (20 mL) and NaOH 2 N (10 mL) was added. The pH was maintained at 13 during 20 min of stirring. The solutions were then acidified with concentrated HCl and the white precipitate was filtered and dried to afford the desired 3-hydroxy-3-(3-hydroxyphenyl)propanoic acid with high purity based on NMR analysis. Yield **4** (1.04 g, 70 %); ^1^H-NMR (D_2_O) δ: 2.78 (m, 2H, -CH_2_-COOH), 5.07 (quartet, 1H, Ph-CH(OH)-), 6.85, 6.91, 6.98, 7.30 (m, 4H, Ph-H). ^13^C-NMR (D_2_O) δ: 46.4, 73.1, 115.7, 117.9, 121.0, 133.1, 147.3, 158.6, 179.0. HRMS-ESI *m*/*z*: calculated for C_9_H_9_O_4_ [M-H]^+^: 181.0506, found: 181.0504.

The same protocol was also used to synthesize ^13^C-HPHPA to afford **5**. Yield **5** (0.97 g, 65 %); ^1^H-NMR (D_2_O) δ: 2.80 (m, 2H, -CH_2_-COOH), 5.08 (quartet, 1H, Ph-CH(OH)-), 6.85, 6.92, 6.98, 7.31 (m, 4H, Ph-H); ^13^C-NMR (D_2_O) δ: 46.07, 72.9, 115.5, 117.8, 121.0, 133.1, 147.3, 158.6, 178.5. HRMS-ESI *m*/*z*: calculated for C_8_[^13^C]H_9_O_4_ [M-H]^+^: 182.054, found: 182.0543.

### 3.2. Materials and Methods for the LC-MS/MS Assay

Optima™ LC/MS-grade formic acid and HPLC-grade water were purchased from Fisher Scientific (Ottawa, ON, Canada). HPLC-grade pyridine and HPLC-grade methanol, 3-nitrophenylhydrazine (3-NPH), 1-ethyl-3-(3-dimethylaminopropyl)carbodiimide (EDC) and butylated hydroxytoluene (BHT) were purchased from Sigma-Aldrich (St. Louis, MO, USA). The pooled urine was purchased from Lee Biosolutions (Maryland Heights, MO, USA). 3-(3-Hydroxyphenyl)-3-hydroxypropionic acid (HPHPA) and ^13^C-labeled HPHPA standards were synthesized and purified in-house. All other organic acid standards including lactic acid, beta-hydroxybutyric acid, alpha-ketoglutaric acid, citric acid, butyric acid, isobutyric acid, propionic acid, p-hydroxyhippuric acid, succinic acid, fumaric acid, pyruvic acid, hippuric acid, methylmalonic acid, homovanillic acid, indole-3-acetic acid, uric acid, and their isotope-labeled standards were purchased from Sigma-Aldrich (St. Louis, MO, USA). All 17 acids along with HPHPA were part of the same assay. Nunc^®^ 96 DeepWell™ plates were purchased from Sigma-Aldrich (St. Louis, MO, USA). Millex-HA Syringe Filter Units (0.22 µm) were purchased from Fisher Scientific (Ottawa, ON, Canada).

Solid chemical standards for all organic acids mentioned above were carefully weighed using a CPA225D semi-micro electronic balance (Sartorius, Bohemia, NY, USA) with a precision of 0.0001 g. Stock solutions, 40 mM for each analyte, were prepared in water by dissolving the weighed solid into specified volumes. Calibration curve solutions were prepared by mixing and diluting the stock solutions with water to generate solutions with concentration ranges from 5 to 400 µM for HPHPA (details of the concentration ranges are not provided here for all other organic acids mentioned above except HPHPA). Stock solutions, 10 mM each, of the isotope-labeled compounds were prepared by dissolving the accurately weighed solids in 75% aqueous methanol. A working internal standard solution mixture in 75% aqueous methanol was made by mixing and diluting all the isotope-labeled stock solutions, with a final concentration of 160 µM for ^13^C-HPHPA. All standard solutions were then stored at −80 °C until further use.

To measure the concentrations of HPHPA in different human urine samples, we used previously collected human urine from 10 healthy volunteers. The study was conducted in compliance with the ethical standards of, and was approved by, the University of Alberta’s HREB biomedical ethics committee (Pro00074045) on 3 October 2017. The urine samples were thawed on ice and filtered with a 0.22 µm Millex-HA syringe filter unit before any further sample preparation. For urine analysis, 10 μL of the ISTD (isotopic standard) mixture solution and 10 μL of the samples were pipetted directly into each spot in a Nunc^®^ 96 DeepWell™ plate. Thirty microliters of 75% aqueous methanol was then added to each well, followed by the addition of 25 μL each of the following three solutions: 3-NPH (3-nitrophenylhydrazine, 250 mM in 50% aqueous methanol), EDC (1-ethyl-3-(3-dimethylaminopropyl)carbodiimide, 150 mM in methanol), and pyridine (7.5% in 75% aqueous methanol). The whole plate was then shaken at room temperature to allow the derivatization reagents to react for 2 h. After derivatization, 350 μL of water and 25 μL of BHT solution (2 mg/mL of butylated hydroxytoluene, in methanol) were added to each plate well to dilute and stabilize the final solutions. Ten microliters of each solution was injected for LC-MS/MS analysis.

Online LC-MS/MS was performed using an Agilent 1100 series HPLC system (Agilent, Palo Alto, CA, USA) equipped with an Agilent reversed-phase Zorbax Eclipse XDB C18 column (3.0 mm × 100 mm, 3.5 μm particle size, 80 Å pore size) coupled to a Phenomenex (Torrance, CA, USA) SecurityGuard C18 pre-column (4.0 mm × 3.0 mm) connected with an AB SCIEX QTRAP^®^ 4000 mass spectrometer (SCIEX, Concord, ON, Canada). The controlling software for the LC-MS system was Analyst^®^ 1.6.2. For the HPLC chromatography, solvent A was 0.01% (*v*/*v*) formic acid in water, and solvent B was 0.01% (*v*/*v*) formic acid in methanol. The solvent gradient profile was as follows: *t* = 0 min, 30% B; *t* = 1.5 min, 30% B; *t* = 12.5 min, 85% B; *t* = 12.51 min, 100% B; *t* = 13.51 min, 100% B; *t* = 13.6 min, 30% B and finally maintained at 30% B for 6.4 min. The column oven was set to 40 °C. The flow rate was 300 μL/min, and the sample injection volume was 10 μL. The MS instrument was set to a negative electrospray ionization mode with multiple reaction monitoring. The IonSpray voltage was set to −4500 volts and the temperature was set to 400 °C. The curtain gas (CUR), ion source gas 1 (GAS1), ion source gas 2 (GAS2) and collision gas (CAD) were set at 20, 30, 30, and medium, respectively. The entrance potential (EP) was set to −10 V. Additionally, the declustering potential (DP), collision energy (CE), collision cell exit potential (CXP), MRM Q1 and Q3 were optimized and set individually for each analyte and each corresponding isotopically-labeled internal standard. In particular, quantification was performed using MRM transition *m*/*z* 316.2→194.2 (DP = −70 V, CE = −20 V, and CXP = −9 V) for HPHPA and *m*/*z* 317.1→195.0 (DP = −69 V, CE = −18 V, and CXP = −9 V) for ^13^C-HPHPA.

Details regarding the method validation for measuring HPHPA, including calibration regression, accuracy and precision, recovery, LOQ, and LOD are given in the Supplementary Materials.

### 3.3. Materials and Methods for NMR Analysis

K_2_HPO_4_ > 98%, KH_2_PO_4_ > 98%, D_2_O 99.9%, and DSS-d_6_ (2,2-dimethyl-2-silapentane-5 sulfonate) 98% were purchased from Sigma-Aldrich (St. Louis, MO, USA). 2-Chloropyrimidine-5-carboxylic acid 98% was obtained from Ark Pharm, Inc. (Libertyville, IL, USA). HPLC-grade H_2_O was purchased from Thermo Fisher Scientific (Hampton, NH, USA).

To enable NMR collection of the pure HPHPA standards for uploading to the Chenomx library, a “reference” buffer solution was prepared. In particular, this buffer contained 250 mM potassium phosphate (pH 7.0), 5.0 mM 2,2-dimethyl-2-silapentane-5 sulfonate (DSS-d_6_), 5.84 mM, 2-chloropyrimidine-5-carboxylic acid, and D_2_O 54% (*v*/*v*) in H_2_O. To prepare the buffer potassium phosphate dibasic (2.67 g), potassium phosphate monobasic (1.3 g), DSS-d_6_ (0.112 g), and 2-chloropyrimidine-5-carboxylic acid (0.092 g) were dissolved in of 99.8% D_2_O (54 mL). The buffer pH was adjusted to 7.00 using a calibrated electronic pH meter through the dropwise addition of 0.1 M potassium hydroxide or 0.1 M phosphoric acid. After pH calibration, HPLC-grade water was added to bring the final volume to 100 mL. A stock solution of 10.44 mM HPHPA was prepared, and 160 µL was transferred to a clean 1.5 mL microcentrifuge tube. Forty microliters of the above NMR “reference” buffer was added to the 1.5 mL microcentrifuge tube to make up a final sample volume of 200 µL. The solution was transferred to a clean 3 mm NMR tube. The final HPHPA concentration in the tube was 8.352 mM. All spectra were recorded at 298 K on an Avance III HD Bruker (700 MHz) spectrometer operating at 700.32 MHz (Bruker Biospin, Rheinstetten, Germany). The spectrometer was equipped with a cryogenically cooled triple resonance probe (TCI). A standard metnoesy (noesypr1d) pulse sequence was employed to collect all HPHPA reference spectra. A relaxation delay of 1 s, an acquisition time of 4 s, and a mixing time of 100 ms were used. A total of 128 transients were recorded. All spectra were processed using Bruker’s Topspin 3.5 pl 7 software. The concentration of the HPHPA was verified by comparing the integrated area of the HPHPA signals with the integrated area of methyl singlet signal at 0.00 ppm arising from DSS. A single reference NMR spectrum of HPHPA (at 1.0 mM) was collected and uploaded to the Chenomx NMR Suite software (version 8.3 Chenomx, Inc., Edmonton, AB, Canada) spectral library, as per the vendor instructions. This reference spectrum was used to identify and quantify HPHPA in the urine samples.

To enable NMR collection of human urine samples, a second “urine” buffer solution was prepared. This buffer contained 750 mM potassium phosphate (pH 7.0), 5.00 mM 2,2-dimethyl-2-silapentane-5 sulfonate (DSS-d_6_), 5.84 mM, 2-chloropyrimidine-5-carboxylic acid, and D_2_O 54% (*v*/*v*) in H_2_O. To prepare the buffer, potassium phosphate dibasic (8.01 g), potassium phosphate monobasic (3.9 g), DSS-d_6_ (0.112 g), and 2-chloropyrimidine-5-carboxylic acid (0.092 g) were dissolved in 99.8% D_2_O (54 mL). The buffer pH was adjusted to 7.00 using a calibrated electronic pH meter through the dropwise addition of 0.1 M potassium hydroxide or 0.1 M phosphoric acid. After the pH calibration was complete, HPLC-grade water was added to bring the final volume to 100 mL. To prepare the urine samples for NMR collection, 160 µL of urine was pipetted to a clean 1.5 mL microcentrifuge tube. Forty microliters of the urine buffer solution was added to the 1.5 mL microcentrifuge tube to give a final sample volume of 200 µL. The solution was transferred to a clean 3-mm NMR tube and the NMR spectra were collected and analyzed as described above. The concentration of HPHPA was determined by fitting the measured urine spectra with the Chenomx NMR Suite software (version 8.3 Chenomx, Inc., Edmonton, AB, Canada) using the modified spectral library.

## 4. Conclusions

In summary, an ultrasonic-mediated Reformatsky reaction using indium powder was developed to synthesize both unlabeled and ^13^C-labeled HPHPA. This new approach to HPHPA synthesis proved to be faster, simpler, and higher-yielding than the previously published method. In particular, the number of synthetic steps was halved, the required synthesis time was cut by tens of hours, the requirement for protection/deprotection steps was avoided, and the reaction conditions proved to be very mild. The resulting HPHPA product was fully characterized and used to identify and quantify the amount of HPHPA found in multiple human urine samples. The synthesized HPHPA standards were also used to develop protocols for the automated or semi-automated identification and quantification of HPHPA in urine by standard NMR and by LC-MS/MS metabolomic techniques.

To our best knowledge, this is the first report describing the synthesis of a stable isotope ^13^C-labeled form of HPHPA. The availability of ^13^C-labeled HPHPA should allow HPHPA to be more easily quantified via standard MS-based metabolomic studies. Furthermore, it may also be used as a potential isotope tracer for in vivo labeling and metabolic flux analysis. Future studies in our group will be focused on the application of both unlabeled and ^13^C-labeled HPHPA to monitor the onset and progression of neurological disorders associated with autism and schizophrenia.

## Figures and Tables

**Table 1:** Comparison of HPHPA concentration results measured by LC-MS and NMR.

Urine ID	HPHPA Concentrations Measured by LC-MS (µM)	HPHPA Concentrations Measured by NMR (µM)	Concentration Differences between LC-MS and NMR (%)
F1	76.1	78.3	2.75
F2	41.5	39.4	5.40
F3	37.6	38.3	1.70
F4	54.3	56.8	4.32
M1	59.3	59.4	0.13
M2	89.2	88.0	1.36
M3	42.5	39.5	7.59
M4	5.48	5.38	1.95
M5	77.0	71.5	7.69
M6	173	172	0.73

## Supplementary Materials

The following are available online at http://www.mdpi.com/2218-1989/8/4/80/s1. Details of the method validation, Figure S1: Representative ^1^H NMR spectra of HPHPA **4**. The peak assignments are indicated with numbers on the structure, Figure S2: Representative ^13^C-NMR spectra of HPHPA **4**. The peak assignments are indicated with numbers on the structure, Figure S3: Representative ^1^H NMR spectra of ^13^C-labeled HPHPA **5**. The peak assignments are indicated with numbers on the structure, Figure S4: Representative ^13^C-NMR spectra of ^13^C-labeled HPHPA **5**. The peak assignments are indicated with numbers on the structure, Figure S5: HPLC chromatogram of HPHPA **4** with UV absorbance measured at 210 and 254 nm. The retention time of HPHPA was 7.04 min, Figure S6: HPLC chromatogram of ^13^C-labeled HPHPA **5** with UV absorbance measured at 210 and 254 nm. The retention time of ^13^C-labeled HPHPA was 7.06 min, Figure S7: HR-MS-ESI for HPHPA **4** C_9_H_10_O_4_, Figure S8: HR-MS-ESI for ^13^C-labeled HPHPA **5** C_8_(^13^C)H_10_O_4_, Table S1: Percentage area report for HPHPA **4**, Table S2: Percentage area report for ^13^C-labeled HPHPA **5**.

## References

[1] Wishart D.S., Knox C., Guo A.C., Eisner R., Young N., Gautam B., Hau D.D., Psychogios N., Dong E., Bouatra S. (2009). HMDB: A knowledgebase for the human metabolome. Nucleic Acids Res..

[2] Wishart D.S., Jewison T., Guo A.C., Wilson M., Knox C., Liu Y., Djoumbou Y., Mandal R., Aziat F., Dong E. (2013). HMDB 3.0—The Human Metabolome Database in 2013. Nucleic Acids Res..

[3] de Angelis M., Francavilla R., Piccolo M., De Giacomo A., Gobbetti M. (2015). Autism spectrum disorders and intestinal microbiota. Gut Microbes.

[4] Keşli R., Gökçen C., Buluǧ U., Terzi Y. (2014). Investigation of the relation between anaerobic bacteria genus clostridium and late-onset autism etiology in children. J. Immunoass. Immunochem..

[5] Shaw W. (2010). Increased urinary excretion of a 3-(3-hydroxyphenyl)-3-hydroxypropionic acid (HPHPA), an abnormal phenylalanine metabolite of *Clostridia* spp. in the gastrointestinal tract, in urine samples from patients with autism and schizophrenia. Nutr. Neurosci..

[6] Xiong X., Liu D., Wang Y., Zeng T., Peng Y. (2016). Urinary 3-(3-Hydroxyphenyl)-3-hydroxypropionic Acid, 3-Hydroxyphenylacetic Acid, and 3-Hydroxyhippuric Acid Are Elevated in Children with Autism Spectrum Disorders. BioMed Res. Int..

[7] Perez-Pineiro R., Dong Y.W., Wishart D.S. (2009). Solid phase synthesis of acylglycine human metabolites. Bioorg. Med. Chem. Lett..

[8] Armstrong M.D., Shaw K. (1957). The occurrence hydracrylic acid in human urine. J. Biol. Chem..

[9] Shen Z., Zhang J., Zou H., Yang M. (1997). A Novel One-Pot Reformatsky Type Reaction via Bismuth Salt in Aqueous Media. Tetrahedron Lett..

[10] Gabriel T., Wessjohann L. (1997). The Chromium-Reformatsky Reaction: Asymmetric Synthesis of the Aldol Fragment of the Cytotoxic Epothilons from 3-(2-Bromoacyl)-2-oxazolidinones. Tetrahedron Lett..

[11] Schick H., Ludwig R., Schwarz K.-H., Kleiner K., Kunath A. (1993). Novel Synthesis of Tetrasubstituted β-Lactones: The Use of Indium in the Electrochemically Supported Reformatsky Reaction. Angew. Chem. Int. Ed. Engl..

[12] Cahiez G., Chavant P.-Y. (1989). Organomanganese (II) Reagents XX: Manganese Mediated Barbier and Reformatsky Like Reactions an Efficient Route to Homoallylic Alcohols and β-acetoxyesters. Tetrahedron Lett..

[13] Araki S., Butsugan Y. (1992). Enantioseiective Synthesis of β-Hydroxy Esters by indium-induced Reformatsky Reaction. J. Chem. Soc. Perkin Trans. 1.

[14] Fürstner A., Bogdanović B. (1996). New developments in the chemistry of low-valent titanium. Angew. Chem. Int. Ed. Engl..

[15] Fürstner A. (1989). Recent Advancements in the Reformatsky Reaction. Synthesis (Stuttg)..

[16] Lee P.H., Bang K., Lee K., Lee C.-H., Chang S. (2000). In-mediated synthesis of 2-(2-hydroxyethyl)homoallenylsilanes. Tetrahedron Lett..

[17] Li C.J. (1996). Aqueous Barbier-Grignard type reaction: Scope, mechanism, and synthetic applications. Tetrahedron.

[18] Li C.J. (1993). Organic Reactions in Aqueous Media—With a Focus on Carbon-Carbon Bond Formation. Chem. Rev..

[19] Jun C., Chan T. (1999). Organic Syntheses Using Indium-Mediated and Catalyzed Reactions in Aqueous Media. Tetrahedron.

[20] Han B.H., Boudjouk P. (1982). Organic Sonochemistry. Sonic Acceleration of the Reformatsky Reaction. J. Org. Chem..

