# Supplementary material for: A Simple and Convenient Synthesis of Unlabeled and 13C-Labeled 3-(3-Hydroxyphenyl)-3-Hydroxypropionic Acid and Its Quantification in Human Urine Samples
Source: Metabolites. 2018 Nov 21;8(4):80. doi: 10.3390/metabo8040080 (PMC6316614; doi:10.3390/metabo8040080)
Supplement: Supplementary file 1 [file metabolites-08-00080-s001.zip › metabolites-375799-SI.pdf]

Metabolites 2018, 8, 80; doi:10.3390/metabo8040080

Supplementary Materials for:

A simple and convenient synthesis of unlabeled and ^{13}C labeled 3-(3-hydroxyphenyl)-3-hydroxypropionic acid and its quantification in human urine samples

Yeganeh Khaniani, Matthias Lipfert, Dipanjan Bhattacharyya, Rolando Perez Pineiro, Jiamin Zheng and David S. Wishart

Method Validation for HPHPA

Calibration Regression

The calibration regression of our LC-MS organic acid assay was evaluated by using seven analyte standard mixture solutions prepared in water. Among all the analytes, concentrations of HPHPA were 5 μM, 10 μM, 20 μM, 40 μM, 80 μM, 160 μM and 400 μM, respectively. All seven standard mixtures were prepared in triplicate. The ratios of the signal intensity (peak area) of HPHPA to ^{13}C-HPHPA were plotted against the known concentrations of HPHPA to build the calibration curves. Due to the 80 times change between the highest and lowest concentrations of the calibrants, quadratic regression was used instead of linear regression.

Accuracy and Precision

To evaluate accuracy and precision, three different concentration levels (low, medium, high) for all analytes, with HPHPA as one of the analytes, were prepared in triplicate, along with one set of calibration curve standards. All these solutions were measured using the method described above and used to calculate the coefficient of variation (CV) expressed as a percentage.

Recovery

Recovery was assessed by analyzing pooled human urine (purchased from Lee Biosolutions) that had been spiked at three different concentrations for all analytes including HPHPA. Each spiked sample was prepared in triplicate. Aliquots of non-spiked pooled human urine samples were analyzed in parallel for recovery calculations. The recovery was expressed as a percentage and was calculated according to Equation 1 shown below, where C_{SP} is the calculated HPHPA concentration in the spiked pooled urine sample, C_{NS} is the calculated HPHPA concentration in the non-spiked pooled urine sample, and C_A is the theoretical concentration of HPHPA that was spiked into the sample.

$$Recovery\ (\%) = (C_{SP} - C_{NS})/C_A \times 100 \qquad (1)$$

Limits of Detection and Quantification

The limit of detection (LOD) and limit of quantification (LOQ) were calculated by analyzing three replicates of the lowest concentration of HPHPA in the calibration curve according to Equations 2 and 3,

where *SD* is the standard deviation of the response and *Slope* is the slope of the calibration curve of HPHPA.

$$LOD = 3 \times SD/Slope \qquad (2)$$

$$LOQ = 10 \times SD/Slope \qquad (3)$$

NMR spectra

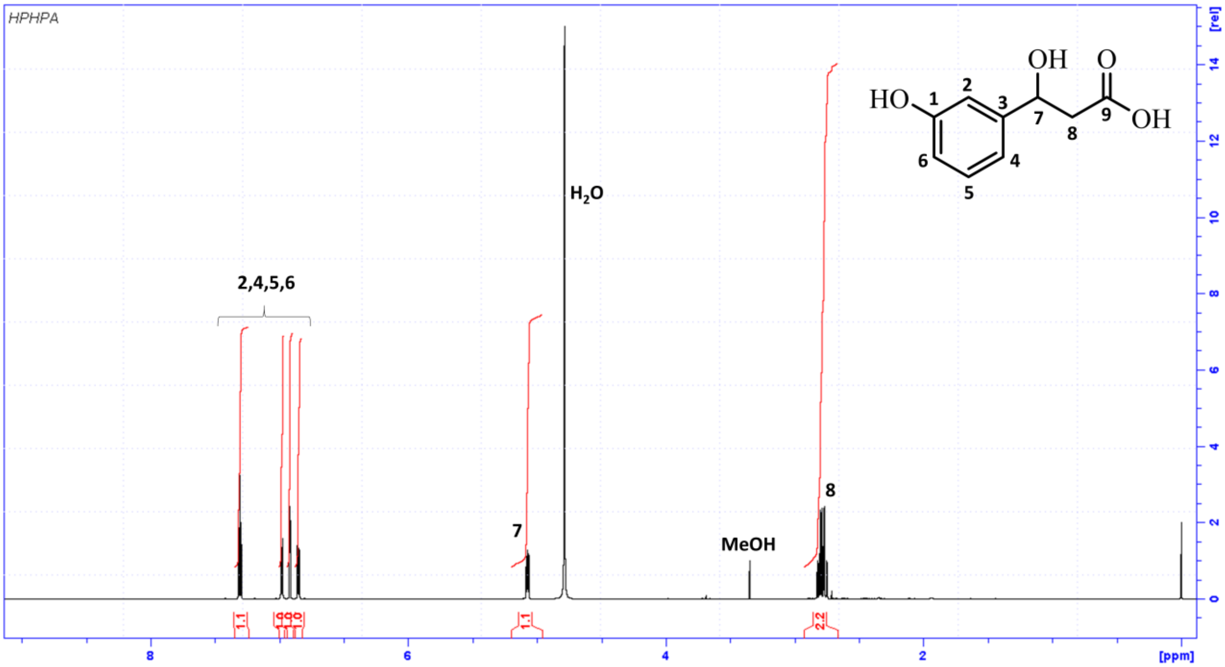

Figure S1. Representative ^{1}H NMR spectra of HPHPA **4**. The peak assignments are indicated with numbers on the structure.

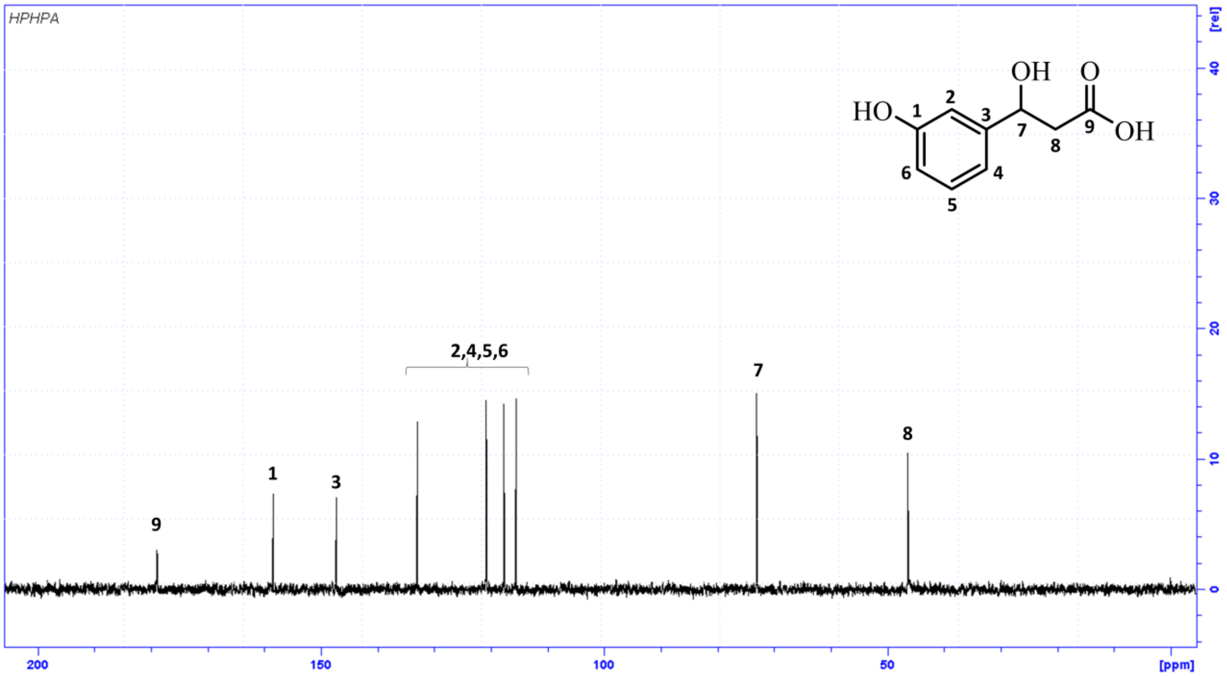

Figure S2. Representative ^{13}C NMR spectra of HPHPA **4**. The peak assignments are indicated with numbers on the structure.

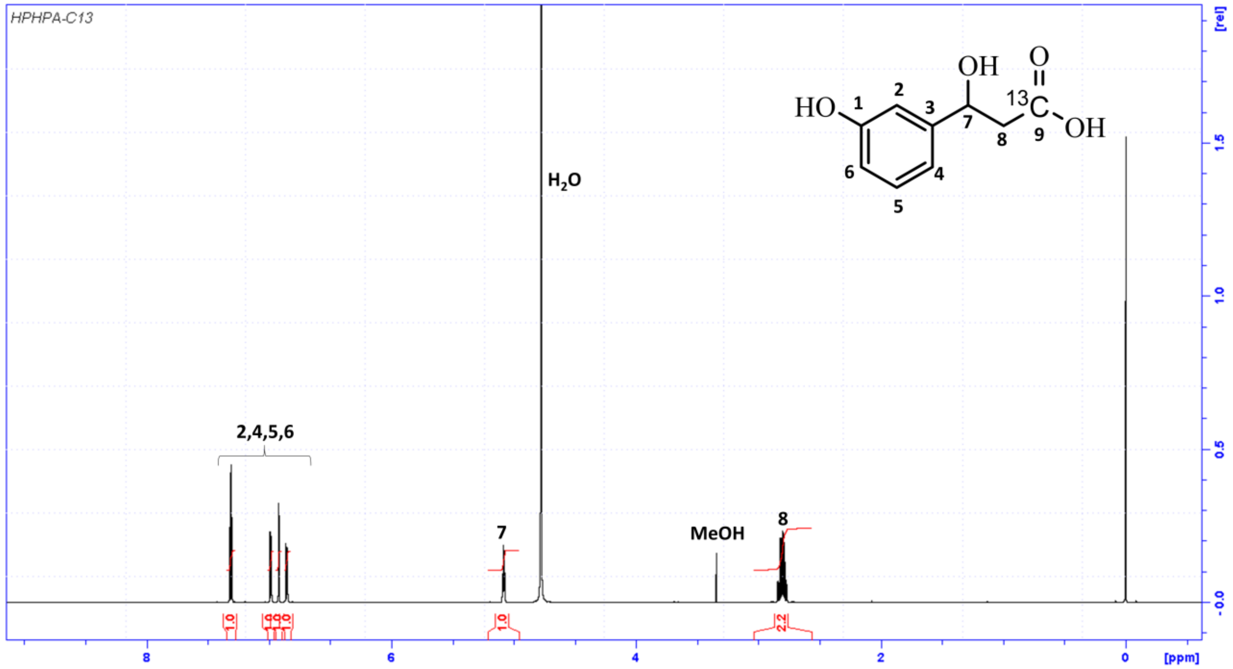

Figure S3. Representative ^{1}H NMR spectra of ^{13}C-labeled HPHPA **5**. The peak assignments are indicated with numbers on the structure.

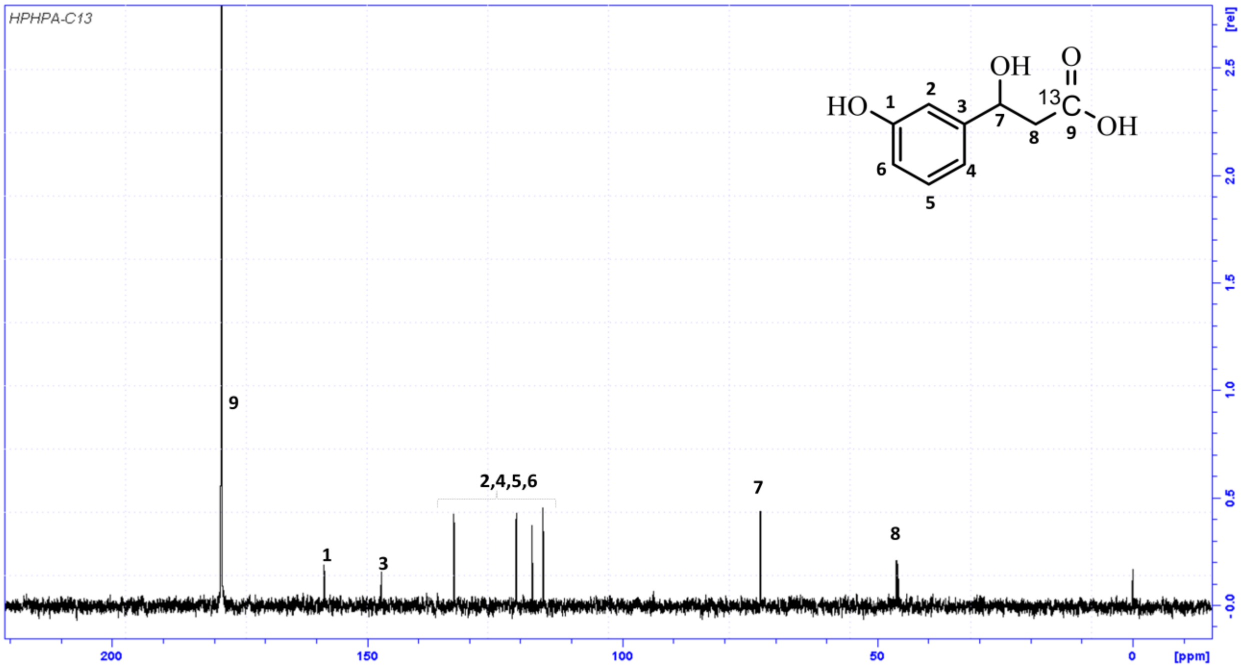

Figure S4. Representative ^{13}C NMR spectra of labeled HPHPA **5**. The peak assignments are indicated with numbers on the structure

HPLC analysis

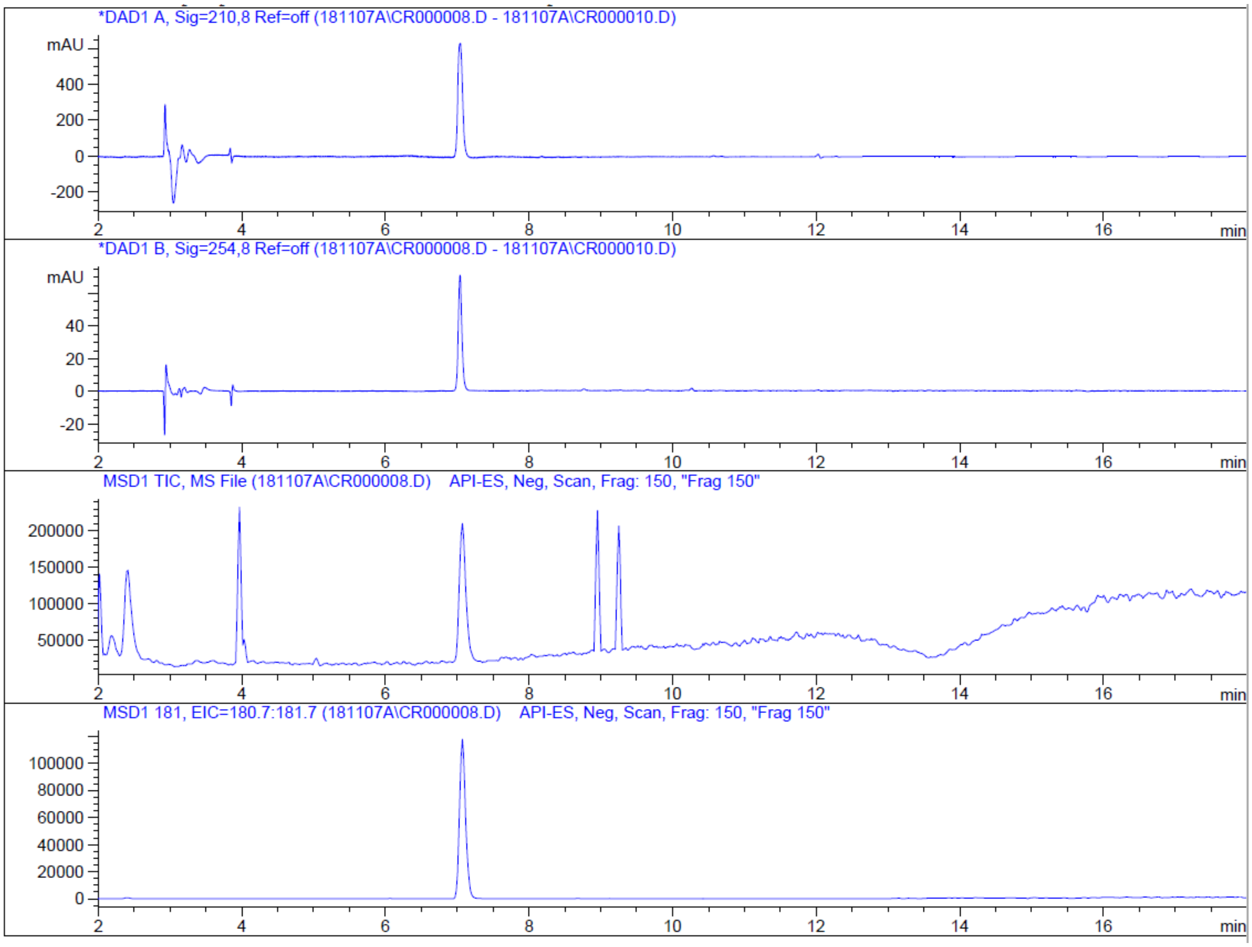

Figure S5. HPLC chromatogram of HPHPA **4** measured at 210 and 254 nm. The retention time of HPHPA was 7.04 min.

Table S1. Area percent report for HPHPA **4**.

Peak No.	Retention time	Type	Width (min)	Area	Height (mAU)	Area %
1	7.044	MM	0.0780	2971.98730	635.02002	98.5876
2	8.173	MM	0.0420	16.45774	6.52595	0.5459
3	8.784	MM	0.0557	8.63714	2.58330	0.2865
4	10.569	MM	0.0511	17.48376	5.70322	0.5800
totals				3014.56594	649.83249	

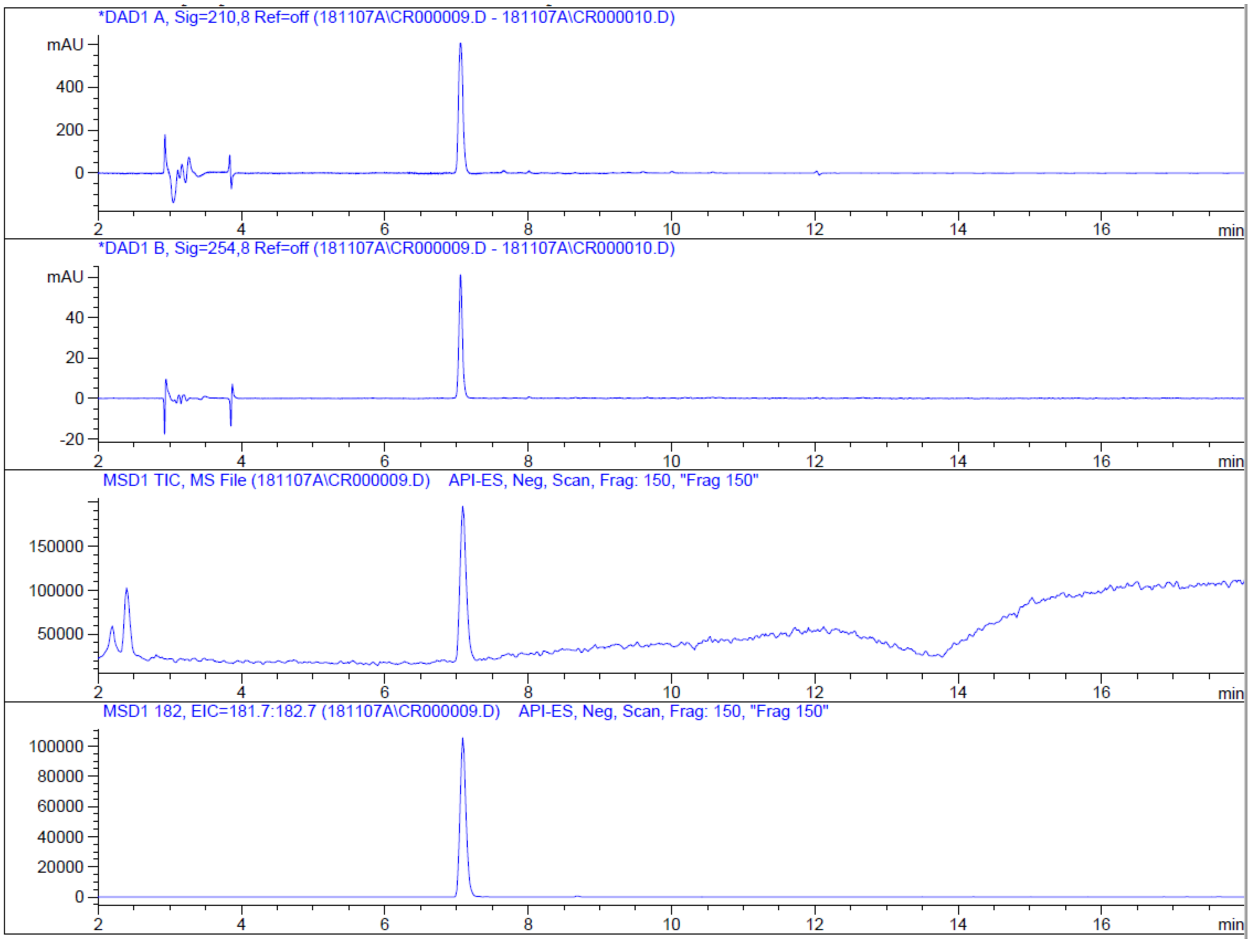

Figure S6. HPLC chromatogram of ^{13}C-labeled HPHPA **5** measured at 210 and 254 nm. The retention time of HPHPA was 7.06 min.

Table S2. Area percent report for HPHPA **5**.

Peak No.	Retention time	Type	Width (min)	Area	Height (mAU)	Area %
1	7.057	MM	0.0579	214.78882	61.87896	96.1949
2	7.659	MM	0.0755	1.22376	0.270229	0.5481
3	8.016	MM	0.0474	2.14914	0.755467	0.9625
4	8.660	MM	0.0463	1.41621	0.509912	0.6343
5	9.662	MM	0.0335	1.05378	0.523878	0.4719
6	10.190	MM	0.0510	1.47027	0.480536	0.6585
7	10.572	MM	0.0453	1.18299	0.435679	0.5298
totals				223.28498	64.85466	

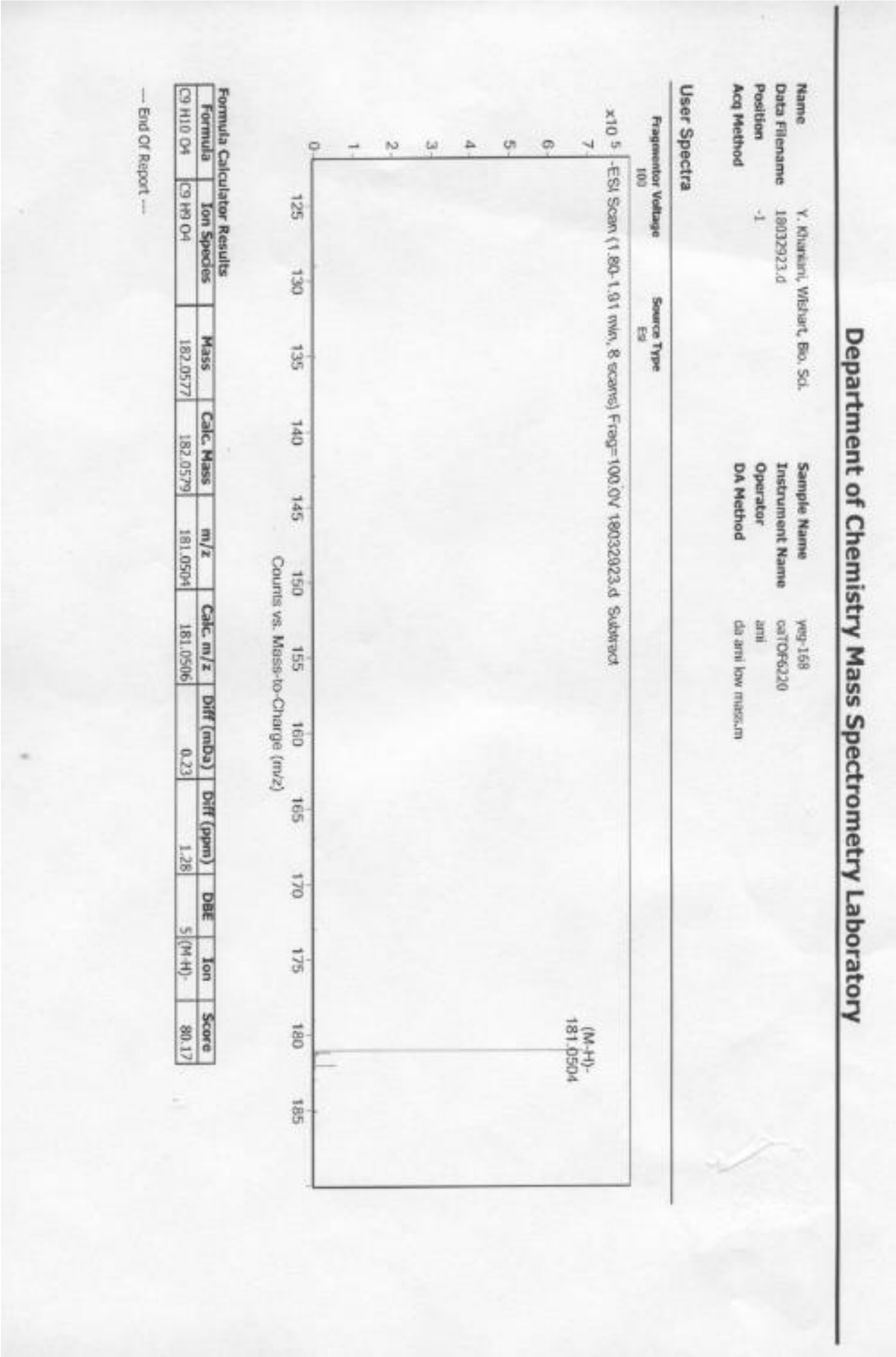

Figure S7. HR-MS-ESI for HPHPA **4** $C_9H_{10}O_4$.

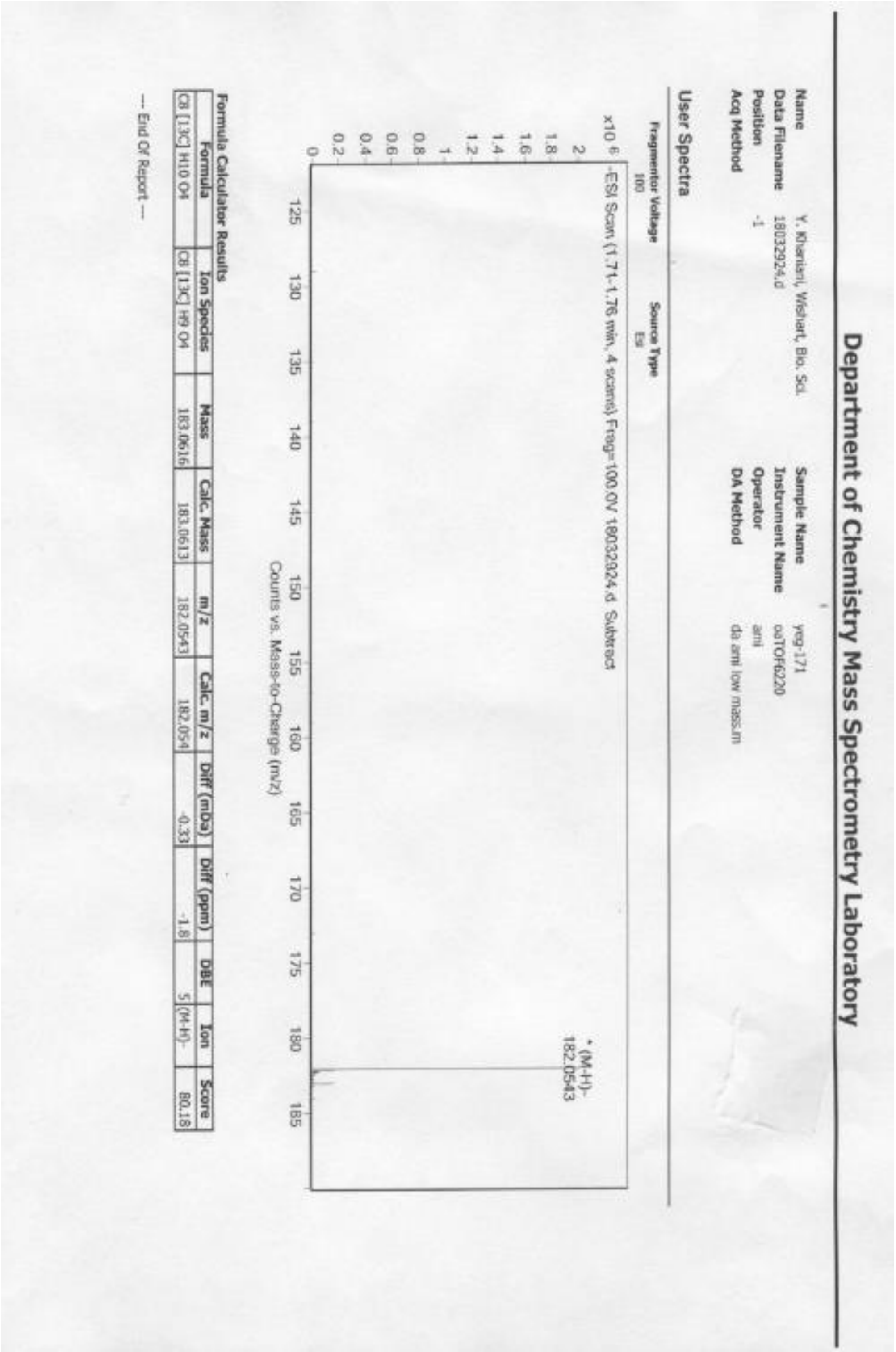

Formula Calculator Results

Formula	Ion Species	Mass	Calc. Mass	m/z	Calc. m/z	Diff (mDa)	Diff (ppm)	DBE	Ion	Score
C8 [13C] H10 O4	C8 [13C] H9 O4	183.0616	183.0613	182.0543	182.054	-0.33	-1.8	5	(M-H)-	80.18

--- End Of Report ---

Figure S8. HR-MS-ESI for ^{13}C-labeled HPHPA **5** $C_8(^{13}C)H_{10}O_4$.
